# Mutually guided light and particle beam propagation

**DOI:** 10.1038/s41598-022-08802-z

**Published:** 2022-03-21

**Authors:** Andres M. Castillo, Prabhat Kumar, Christopher M. Limbach, Kentaro Hara

**Affiliations:** 1 Aeronautics and Astronautics, Stanford University, Stanford, 94305 CA USA; 2 Center for Computational Sciences and Engineering, Lawrence Berkeley National Laboratory, Berkeley, CA 94720 USA; 3 Aerospace Engineering, Texas A&M University, College Station, TX 77843 USA

**Keywords:** Aerospace engineering, Atomic and molecular interactions with photons, Atom optics, Nonlinear optics

## Abstract

The polarizability of atoms and molecules gives rise to optical forces that trap particles and a refractive index that guides light beams, potentially leading to a self-guided laser and particle beam propagation. In this paper, the mutual interactions between an expanding particle beam and a diffracting light beam are investigated using an axisymmetric particle-light coupled simulation. The nonlinear coupling between particles and photons is dependent on the particle beam radius, particle density, particle velocity and temperature, polarizability, light beam waist, light frequency (with respect to the resonance frequency), and light intensity. The computational results show that the maximum propagation distance is achieved when the waveguiding effect is optimized to single-mode operation. The application of the coupled beam propagation as a space propulsion system is discussed.

## Introduction

The ability for light to influence particles through optical forces has been studied since the 1950s^1,2^. With increased experimental work in this field during the 1970s, optical tweezers that trap particles using a light beam enabled developments in a variety of applications^3,4^, including surface imaging or force sensing for biological systems^5^. Optical forces have been used for particle trapping and in sub-Doppler cooling devices that can compress and cool molecules to the order of $\upmu$K^6,7^. The manipulation of light has also been studied and employed, most notably through the use of optical waveguides, which are a critical component of high-speed communication and computing systems^8,9^. Recent experiments and simulations show the capability of beam self-cleaning using a multi-mode fiber, opening the door for further studies in nonlinear interactions relevant to high-power fiber systems^10,11^.

Light and particle beams have been considered for space propulsion systems. For instance, solar sail utilizes radiation pressure exerted by sunlight on spacecraft, which is demonstrated by IKAROS^12^. The discovery of Proxima Centauri b has also triggered research in the area of beamed power propulsion systems^13^. Despite the advantage of being completely external propulsion systems, employing either a laser or particle beam to provide thrust for a spacecraft can be considerably limited due to beam diffraction or diffusion. Consequently, the spacecraft would be accelerated only during the time that the beam is concentrated enough to deliver noticeable momentum transfer, which results in thrust. While degradation of the spacecraft materials by the intense laser and particle beams could pose challenges in realizing beamed-power propulsion systems^14,15^, another fundamental limitation of the technology lies in the propagation distance of the beams.

By tuning the laser frequency near resonance, gaseous particles experience a dipole force that lead to trapping or detrapping of the particles. This has been shown in past experiments, where a converging light beam is used to trap gaseous alkali particles, resulting in the amplification of particle beam density^16–18^. In addition, the atomic polarizability and gas density affects the refractive index of light propagation, which may lead to guiding or defocusing light. Numerical work by Kumar et al. has quantitatively validated the experimental results by Pearson et al.^19^ and reported notable waveguiding in higher particle density cases^20^. These results considered a *converging* light beam that forces the gaseous particles to be trapped within a short propagation distance. However, the ability to self-propagate an initially *diverging* light beam and an initially expanding particle beam has not yet been demonstrated. The tailoring of optical forces and refractive interactions so as to produce self-channeling would represent a significant step forward in the ability to engineer particle-light interactions. For propulsion applications, the key metric of interest is the enhancement of the propagation distance beyond that determined by the Rayleigh criterion for a diffracting Gaussian beam in vacuum.

In this work, a validated cylindrical particle-light coupling simulation is utilized to explore the conditions for mutual guiding of a diffusing particle beam and a diffracting light beam. While the nonlinear physics can be extended to different scales, the parameters for the simulations in the paper are chosen to demonstrate the capabilities of the self-guided particle and light beam as a space propulsion technique. The governing equation for the light beam propagation and the optical forces on the particles are reviewed in “Particle-light coupling physics” section. The pertinent parameters that characterize light and particle beams are in “Parametric study” section. “Simulation results” section presents the optimal configuration and condition for maximizing propagation distance. The optimal configuration demonstrates the superiority of the coupled light-particle beam over the individual beams.

## Particle-light coupling physics

### Governing equation for light beam

As derived from the Maxwell’s equations in previous work^21^, the governing equation for the light beam is the axisymmetric paraxial Helmholtz equation, assuming that the wave propagation in the axial (*z*) direction is dominant over the transverse ($\perp$) direction and consequently employing the slowly varying envelope approximation. This relation is also solved independent of time, which assumes the refractive index varies slowly compared to the speed of light. The evolution of the electric field can be expressed as

1 $$\begin{aligned} \nabla ^2_\perp {\overline{E}}+2ik \frac{\partial {\overline{E}}}{\partial z}+k^2(n^2-1){\overline{E}}=0, \end{aligned}$$

where ${\overline{E}}$ is the slowly varying amplitude of the complex electric field, $k=2\pi /\lambda$ is the vacuum wave number, $\lambda$ is the wavelength, and *n* is the refractive index. The refractive index can be modelled as $n=(1+\chi )^{1/2}$, where $\chi =N\alpha /\varepsilon _0$ is the electric susceptibility for a rarefied medium, *N* is the number density, $\alpha$ is the complex polarizability, and $\varepsilon _0$ is the vacuum permittivity. The real part of the propagation constant, or equivalently, the refractive index, is associated with changes in the phase speed of the wave and leads to defocusing or guiding of the light. The imaginary part corresponds to attenuation by absorption and emission.

### Optical forces on particles

The two forces that the light beam imposes on the particles are the dipole and scattering force, which depend on the real ($\alpha '$) and imaginary ($\alpha ''$) components of the atomic polarizability, respectively. Assuming the laser frequency is near atomic resonance, the polarizability for a two-level atom can be written as^16,22–24^

2 $$\begin{aligned} \alpha =\alpha '+i\alpha ''=\frac{4\pi \varepsilon _0}{1+p(f)}\frac{\lambda ^3_0}{16\pi ^3}\frac{\gamma _N}{2}\frac{-\Delta f+i(\frac{\gamma _N}{2})}{(\Delta f)^2+\frac{\gamma ^2_N}{4}}, \end{aligned}$$

where $\lambda _0$ is the resonance wavelength, $\gamma _N$ is the natural line width, $\Delta f=f_{dop}-f_0$ is the laser detuning from particle resonance, $f_{dop}=f-v_b/\lambda$ is the Doppler-shifted laser frequency, $f=c/\lambda$ is the laser frequency, *c* is the speed of light, $v_b$ is the particle velocity, and $f_0=c/ \lambda _0$ is the atomic resonance frequency. Here, *p*(*f*) is the saturation parameter, which is expressed as

3 $$\begin{aligned} p(f)=\frac{I}{I_{\mathrm{sat}}}\frac{\gamma ^2_N/4}{(\Delta f)^2+\gamma ^2_N/4}, \end{aligned}$$

where $I=\frac{1}{2}c\varepsilon |E|^2$ is the laser intensity, $\varepsilon$ is the permittivity of the medium, and

4 $$\begin{aligned} I_{\mathrm{sat}}=\frac{2\pi ^2hf_{\mathrm{dop}}^3\gamma _N}{c^2} \end{aligned}$$

is the saturation intensity, with *h* being the Planck constant.

The real component of the atomic polarizability affects the dipole force through the following relation:

5 $$\begin{aligned} {\mathbf {F}}_{\mathrm{dip}}=\frac{1}{2c\varepsilon _0}\alpha '\nabla I=-\nabla U, \end{aligned}$$

where $U=-\alpha 'I/(2c\varepsilon _0)$ is the optical dipole potential^25^. Here, $\varepsilon \approx \varepsilon _0$ for rarefied media. The optical dipole force works to push and trap the particles towards the centerline of the beam, if the light intensity is largest at the centerline of the beam and the real component of the polarizability is positive. As can be seen from Eq. (2), this corresponds to a negative laser detuning, i.e., red detuning. Furthermore, for a particle beam to be effectively trapped by the light beam, $U>k_BT_\perp$ must be satisfied, where $k_B$ is the Boltzmann constant and $T_\perp$ is the perpendicular particle temperature^21^.

On the other hand, the scattering force is linked with the imaginary component of polarizability as

6 $$\begin{aligned} {\mathbf {F}}_{\mathrm{scat}}=\frac{k_0}{c\varepsilon _0}\alpha ''I\hat{{\varvec{k}}}, \end{aligned}$$

where ${\hat{{\varvec{k}}}}$ is the direction at which the scattering force is applied to. During a scattering event, a particle absorbs and then reemits a photon, essentially receiving two adjustments in momentum. The scattering force is directed along the light wave vector to model an absorption event. Assuming that a photon can then leave the particle in any direction via spontaneous emission after the light is absorbed by the particle, the emission component of the scattering force leads to isotropic scattering of the particle. In order to account for both processes, the computational model applies the scattering force on a particle in the direction of the light wave vector and in a random direction to represent absorption and emission events, respectively.

**Figure 1:**
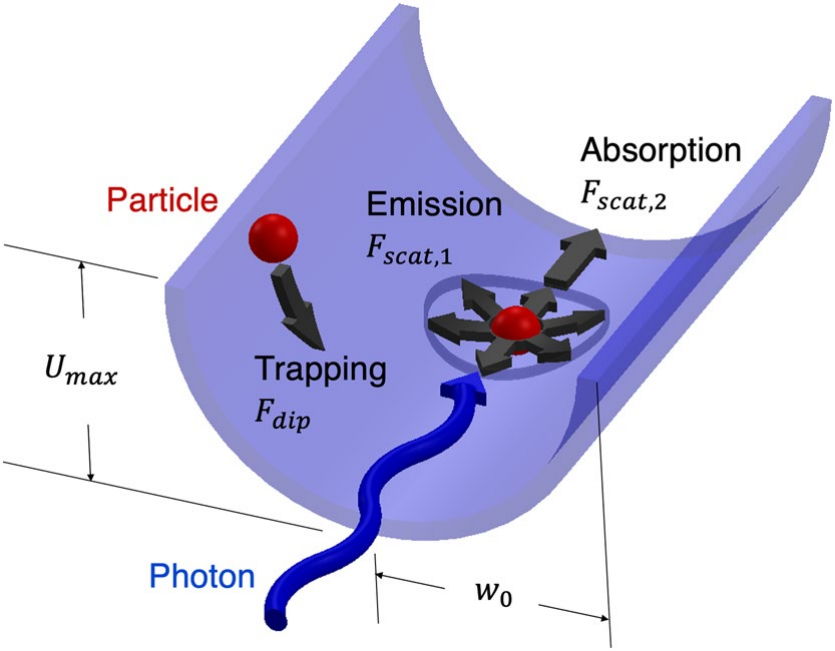
Schematic of the optical forces that the particles experience in the region of large light intensity gradient. $U_{\mathrm{max}}=\alpha 'I_0/(2c\varepsilon _0)$ is the maximum potential amplitude that is dependent on the maximum light intensity, $I_0$, and the beam radius, $w_0$. Note the scattering force has two contributions: absorption in the direction of the incoming photon and spontaneous emission resulting in a momentum change in a random direction.

The two optical forces on individual particles are visualized in Fig. 1. Considering the dependence of the dipole force on the gradient of the laser intensity, particle trapping can be represented through the amplitude of a potential well, $U_{\mathrm{max}}=\frac{\alpha 'I_0}{2c\varepsilon _0}$, where $I_0$ is the centerline intensity. This simplified schematic assumes the potential structure remains constant along the axis of propagation. In reality, the shape of the well would evolve in the axial direction. It can be seen that not only does the strength of trapping depend on the real component of polarizability and peak intensity, but the initial light beam waist, $w_0$, also plays a role by directly determining the shape of the potential well. The depiction of the scattering force demonstrates how the particles can be detrapped from the potential well, particularly due to the random kick from the emission process.

**Figure 2:**
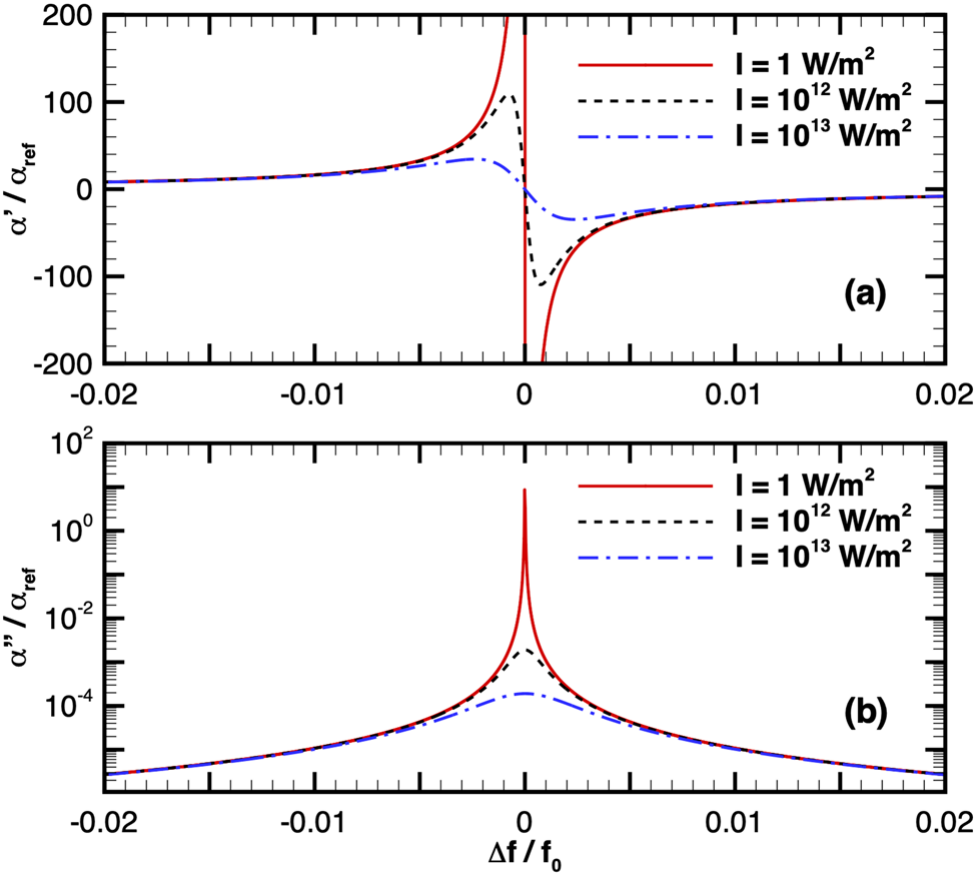
(**a**) Real and (**b**) imaginary component of lithium polarizability over a range of light detuning and for a few light intensities, *I*. Polarizability is calculated using the resonance wavelength of the $D_2$ line of ${}^6$Li, $\lambda _0=670.977$ nm and spontaneous emission rate, $\gamma _N=5.9\times 10^{6}$ Hz. Note that $\alpha _{\mathrm{ref}}=2.704\times 10^{-39}$ F$\mathrm{m}^2$.

From Eqs. (5) and (6), it is readily apparent that the atomic polarizability is a key element in both the dipole and scattering forces. Equation (2) illuminates how the choice of laser frequency with respect to resonance determines the real and imaginary polarizability values. Figure 2 shows the real and imaginary components of polarizability of lithium as a function of Doppler-shifted laser detuning from resonance. Polarizability is calculated using the resonance wavelength of the $D_2$ line of ${}^6$Li, $\lambda _0=670.977$ nm, spontaneous emission rate, $\gamma _N=5.9\times 10^{6}$ Hz, and several different light intensities. Note that the saturation intensity is 66.9 W/$\mathrm{m}^2$ near resonance, the laser detuning is normalized by the particle resonance frequency, and the polarizability is normalized by a reference value, $\alpha _{\mathrm{ref}}=2.704\times 10^{-39}$ F$\mathrm{m}^2$, which is the far-off resonance, frequency-independent polarizability of lithium found experimentally and theoretically^26,27^. These results imply that the laser should be tuned close to resonance to strengthen the dipole force, but not so close that the scattering force dominates. Through the saturation parameter, the light intensity functions to reduce both components of polarizability, with the most notable impact occurring within a close range of particle resonance. It is also important to note that higher light intensity leads to a larger reduction of polarizability values over a wider range in detuning from resonance.

## Parametric study

For the particle beam, the governing features are the type of particle, beam shape, temperature, initial radius, axial drift velocity, and the injected particle density. In this study, the particle type and drift velocity are chosen to be lithium and 0.1*c*, respectively, based on previous work considering an application of the coupled beam system for a flyby mission to Proxima Centuari b. In addition to mission-related considerations, selecting alkali metals for the particle type provides the advantage of modelling the polarizability using a two-level atomic model. Furthermore, the particle beam shape is constructed to have a uniform injection density in the radial direction, i.e., the particle density at the injection plane is constant within the beam radius.

For the light beam, the characteristic features are the injection power, beam waist, and frequency (or equivalently, wavelength). A potential additional feature is the profile of the light beam. An axisymmetric Gaussian laser beam ($\rm TEM_{00}$ mode) is chosen for this study. Lastly, The particle beam radius is chosen to be equivalent to the light beam waist to maximize the light-particle interaction^20^.

### Theoretical trapping and vacuum propagation

As will be seen in the following section, optical waveguide theory suggests that a laser could be effectively guided given a collimated particle beam of a certain density^28^. Considering a constant laser power, the laser beam waist determines the light intensity (i.e., power density): $P_0 = \pi w_0^2I_0 / 2$, where $P_0$ is the light power, assuming a Gaussian light beam. While light intensity is proportional to the trapping potential, $U_{\mathrm{max}}$, the saturation parameter (Eq. 3) becomes appreciable above a certain range of light intensity and causes the polarizability to decrease. In addition, the larger the light intensity, the larger the scattering force, which may result in larger loss mechanism for particles. Thus, it can be expected that there is an optimal light intensity that maximizes trapping and minimizes scattering.

**Figure 3:**
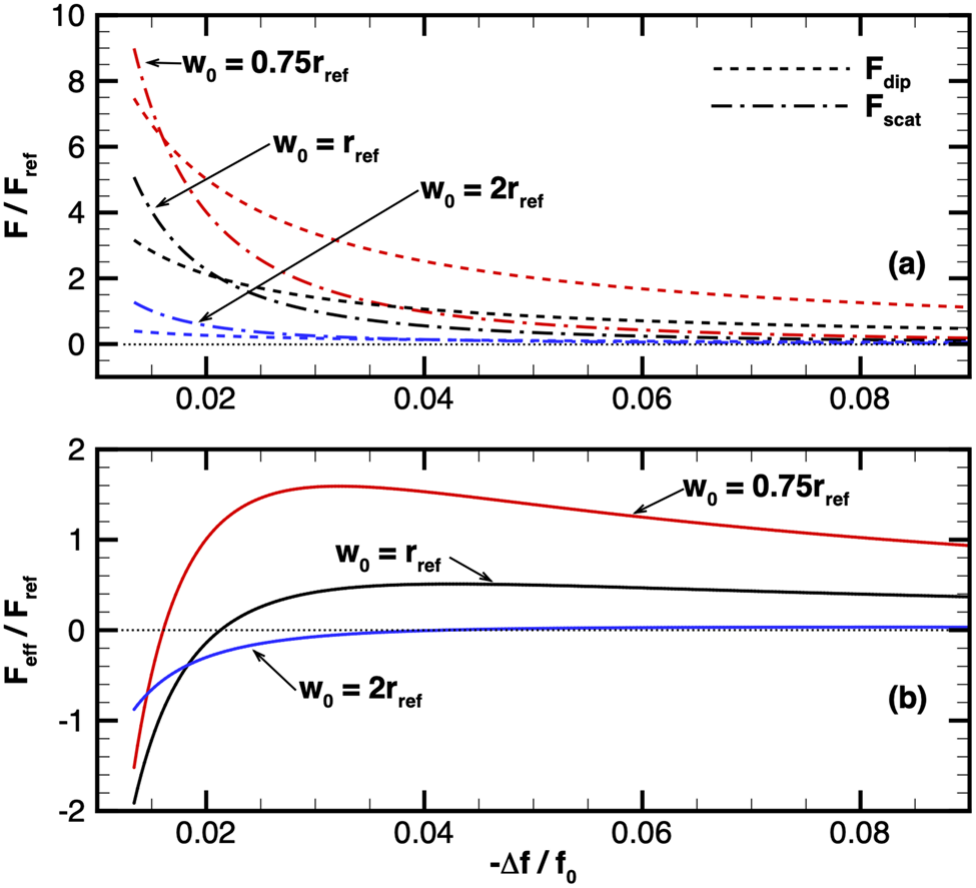
Influence of beam waist and detuning on trapping. (**a**) Both optical forces and (**b**) the effective trapping force over a range of detuning and for a few different beam waists. Light power is 2.2 TW. Note the light detuning below resonance is Doppler-shifted considering a particle beam velocity of 0.1*c*, $F_{\mathrm{ref}}=2.76\times 10^{-24}$ N, and $r_{\mathrm{ref}}=1$ m.

It was proposed in Ref.^21^ that one condition for particle trapping is $U \gg k_B T_\perp$ in the absence of particle-photon scattering. To quantitatively assess the impact of the laser detuning and beam waist on particle trapping in the presence of particle-photon scattering, the effective particle trapping can be approximated, to first order, by comparing the magnitude of the dipole force (resulting in trapping) and the magnitude of the scattering force (particularly due to the spontaneous emission), as shown in Fig. 1. The dipole force is approximated as $F_{\mathrm{dip}} \approx \frac{\alpha 'I_0}{2w_0c\varepsilon _0}$, and the scattering force is approximated as $F_{\mathrm{scat}} \approx \frac{k_0\alpha ''I_0}{ c\varepsilon _0}$. Both $F_{\mathrm{dip}}$ and $F_{\mathrm{scat}}$ have a nonlinear relation with the beam waist because $I_0$ is a function of $w_0$ and the polarizability can be affected by the light intensity, considering a constant light power, $P_0$. Figure 3a illustrates the approximations for both of these forces over a range of detuning below the particle resonance for a few different beam waists. At light frequency near particle resonance, the scattering force dominates over the dipole force for larger $w_0$ because $F_{\mathrm{dip}} \propto w_0^{-3}$ and $F_{\mathrm{scat}} \propto w_0^{-2}$. Consequently, smaller beam waists result in the dipole force dominating over scattering force in a wider range of light frequencies. The beam waists are normalized by a reference radial length, $r_{\mathrm{ref}}=1$ m, and the forces are normalized by a reference dipole force, $F_{\mathrm{ref}}=\frac{\alpha _{\mathrm{ref}}I_{\mathrm{ref}}}{2c\varepsilon _0r_{\mathrm{ref}}}$, where $I_{\mathrm{ref}}$ is the reference light intensity. For the reference values we consider a trapping potential that retains approximately $95\%$ of the particles in the Gaussian beam, i.e., $U_{\mathrm{ref}}=2k_B T_{\mathrm{ref}}$, where $T_{\mathrm{ref}}=0.1$ K is chosen to be the particle temperature. The relation from Eq. (5) yields $I_{\mathrm{ref}}=2U_{\mathrm{ref}}c\varepsilon _0/\alpha _{\mathrm{ref}}$, which results in $F_{\mathrm{ref}}=2.76\times 10^{-24}$ N.

The overall strength of particle trapping is quantified using the effective trapping force introduced as

7 $$\begin{aligned} F_{\mathrm{eff}}=F_{\mathrm{dip}}-F_{\mathrm{scat}}\approx \frac{I_0}{c\varepsilon _0}\left( \frac{\alpha '}{2w_0}-k_0\alpha ''\right) . \end{aligned}$$

Note that $F_{\mathrm{eff}}$ is chosen as a metric of interest as opposed to the ratio between the dipole and scattering force to show the absolute force exerted on particles. Figure 3b shows the effective trapping force near the resonance frequency. This result further illustrates the advantage of smaller beam waists to produce effective trapping as $F_{\mathrm{eff}}>0$ for a wide range of frequencies. Most importantly, Fig. 3b identifies the light frequency that *maximizes* the effective trapping force for a given beam waist. Thus, once the beam waist is chosen, the optimal detuning frequency can be readily determined.

**Figure 4:**
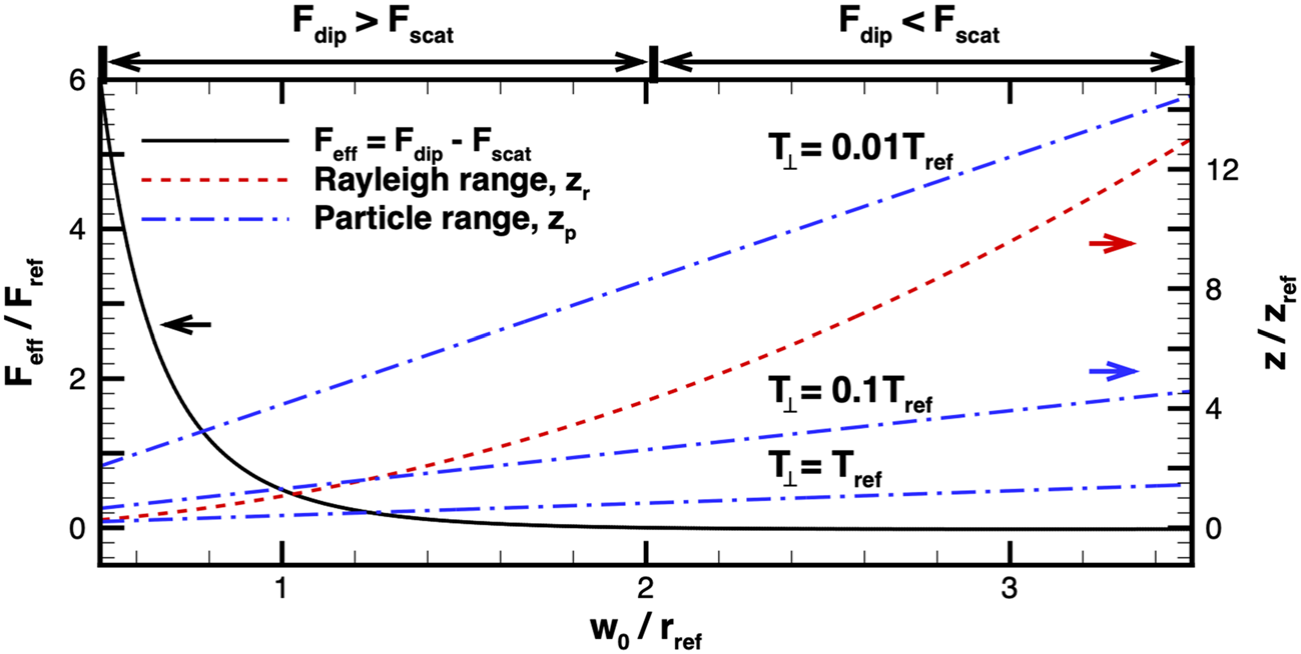
Relevant factors affected by the light beam waist, which is assumed to be equal to the particle beam radius. The left axis corresponds to the effective trapping force (Eq. 7) and the right axis corresponds to the light and particle characteristic lengths $z_r$ (Eq. 8) and $z_p$ (Eq. 9), respectively. Different values for the particle beam temperature are shown for $z_p$. The light power and detuning frequency are 2.2 TW and $-19$ THz, respectively. Also note that $T_{\mathrm{ref}}=0.1$ K, $z_{\mathrm{ref}}=4.68\times 10^{6}$ m, $r_{\mathrm{ref}}=1$ m, and $F_{\mathrm{ref}}=2.76\times 10^{-24}$ N are considered for normalization.

Although a smaller beam waist is preferred to maximize the effective trapping force (i.e., the dipole force minus the scattering force) as shown in Fig. 3, the vacuum propagation distance of the light and particle beams increase with beam waist. The propagation distance of the light beam in vacuum is characterized by the Rayleigh range:

8 $$\begin{aligned} z_r=\frac{\pi w^2_0}{\lambda }, \end{aligned}$$

which describes the axial distance that the laser beam would double its cross-section size from the focus position^29^. An analogous relation for the particle beam propagation distance can be introduced as

9 $$\begin{aligned} z_p = w_0v_b\sqrt{\frac{m}{2k_BT_\perp }}, \end{aligned}$$

where *m* is the particle mass. Here, we call this quantity the particle propagation range. Similar to the Rayleigh range, the particle beam shows a longer propagation in vacuum with a larger beam waist and a smaller gas temperature, assuming that the light beam waist is equal to the particle beam radius. While a smaller beam waist is preferable to increase the effective trapping force, a larger beam waist is desirable to extend the characteristic lengths of the individual beams.

Figure 4 shows how the effective trapping force (left axis) and characteristic lengths (right axis) vary with light beam waist. The laser power, detuning frequency, and particle velocity are assumed constant at 2.2 TW, $-19$ THz, and 0.1*c*, respectively, and $z_{\mathrm{ref}}=\pi r_{\mathrm{ref}}^2/\lambda _0$ is used to normalize the characteristic lengths. Furthermore, the light beam waist is assumed to be equal to the particle beam radius^30^. The effective trapping force, $F_{\mathrm{eff}}$, is inversely proportional to the beam waist as shown in Eq. (7). However, the Rayleigh range, $z_r$, is quadratically proportional to the beam waist (see Eq. 8) and the particle propagation range, $z_p$, is linearly proportional to the beam waist (see Eq. 9). Here, three different radial temperatures of the particle beam are considered to illustrate that the colder the temperature, the less diffusion of particle beam, and hence the longer the particle beam propagates. This result can identify three approximate regions for the beam waist. For a beam waist of less than 0.8$r_{\mathrm{ref}}$, the trapping force is favorable, but $z_p$ and $z_r$ are both small. On the other hand, if the waist is larger than 2$r_{\mathrm{ref}}$, the effective trapping force becomes negative, which indicates the scattering process dominates over the dipole force. The characteristic lengths of light and low temperature particle beam may be large, but the scattering will limit the propagation distance of the beam. Thus, an intermediate beam waist that achieves notable effective trapping force and characteristic lengths is optimal to maximize the *absolute* distance of the coupled beam propagation. It is also important to note how the difference between $z_r$ and $z_p$ influences the effectiveness of trapping or guiding. A large difference between these two lengths would correspond to significantly different divergence angles in the light and particle beams, which consequently may reduce the particle-light interactions in the outer regions of the beams. It was hypothesized in Kumar et al. that the nonlinear coupling of particle and light beams is achieved when the two beams geometrically overlap^20^.

Optimization of the beam propagation depends on multiple parameters of both the light and particle beams. The axisymmetric particle-light coupling simulation allows for the parametric study to investigate the optimal conditions of the self-guided propagation. In the remainder of the paper, the two main parameters of interest are identified as the gas particle density and light intensity, once the beam waist and light frequency are determined to maximize the effective trapping force and the propagation distance. A beam waist of 1 m is chosen to serve as a compromise between the vacuum propagation of the particle and light beams and the effective trapping force, as can be seen in Fig. 4. This beam waist maximizes the effective trapping force at a detuning frequency of 19 THz below resonance ($-\Delta f/f_0=0.042$), as can be seen in Fig. 3. A more rigorous optimization of the light beam waist and frequency via a parametric study using the coupled beam simulation is reserved for future work. Lastly, although experimental work using a magneto-optical-trap have shown the capability of producing a cold, lithium atom beam with transverse temperatures around 0.001 K^31^, a gas temperature of 0.1 K is assumed taking into account the uncertainties of beam formation in the space environment. Note that a gas temperature of 0.1 K and beam waist of 1 m results in $z_p/z_r = 0.389$ at the injection plane.

### Optical waveguide theory

The most relevant real-world example of light guiding is communication and computing systems that use optical waveguides^8^. An optical waveguide, such as an optical fiber, is a structure usually composed of a cylindrical glass core with index of refraction, $n_1$, surrounded by an annular cladding material with index of refraction, $n_2$. Light enters through the core and is maintained within the waveguide due to refraction at the core/cladding interface. For certain waveguide conditions, the profile of the incoming light can be reshaped into specific modes. To determine what mode(s) propagate, a V-parameter is given as,

10 $$\begin{aligned} V = \frac{2\pi a\sqrt{n^2_1-n^2_2}}{\lambda }, \end{aligned}$$

where *a* is the radius of the waveguide core^28^. For a step-index fiber, single mode propagation occurs at $V = 2.405$, which is the first zero of the Bessel function that characterizes the modes in the waveguide. This relation can be applied to the coupled light-particle beam by considering the particle beam to be the core of the waveguide and the outside vacuum as the cladding, i.e., $n_2^2=1$, $a=w_0$, and expressing the index of refraction in terms of gas properties, $n^2_1=1+N\alpha '/\varepsilon _0$. The V-parameter for the light-particle beam coupling can therefore be given from Eq. (10) as,

11 $$\begin{aligned} V = \frac{2\pi w_0}{\lambda } \sqrt{\frac{N \alpha ' }{ \varepsilon _0}}. \end{aligned}$$

Once the polarizability, light wavelength, and beam waist are set, the number density of gaseous particles can be determined to achieve a given V-parameter. In this study, the number density at which a single mode propagation occurs, i.e., $V=2.405$, is defined as the critical particle density, $N_{0}$.

The single mode waveguides operate at the lowest mode, which yields axisymmetric modes. It can therefore be considered that the coupled beam will be axisymmetric even if the initial light or particle beam may be azimuthally nonuniform. When the number density of the particle beam is larger than the critical particle density, the V-parameter exceeds 2.405, leading to the generation of multimode propagation^10,11^, which can result in the generation of azimuthal modes. In addition, it is known from waveguide theory that modal dispersion limits the distance capabilities of multimode optical fibers^32^. Therefore, we hypothesize that axisymmetric mode propagation leads to the most effective coupled beam for the space propulsion application below. In this study, we vary the number density of the particle beam from $0.2 N_0$ to $6 N_0$, assuming axisymmetric (single mode) beams for the space propulsion application. Investigation of the non-axisymmetric beams, particularly for multimode regime, requires a three-dimensional simulation and is reserved for future work.

## Simulation results

Equation (1) is discretized using the first-order backward Euler method and solved as a tridiagonal matrix problem. To minimize the reflection of the laser beam at the domain boundary, a transparent boundary condition is used. This boundary condition was developed in Ref. ^20^ to accommodate wide-angle propagation waves.

The particles are first introduced into the domain within the beam radius by sampling velocities from a shifted Maxwellian distribution, with the drift velocity in the axial direction. To reduce numerical noise due to the particle dynamics, a variable grid step size is used in the radial direction that maintains equal node volumes^33^. Thus, the radial cell size decreases from the center while the axial cell size is uniform. The particle positions and velocities are updated using a leap frog method. Particle-particle collisions are accounted for using a Direct Simulation Monte Carlo (DSMC) algorithm with the No-Time-Counter Method, as described in Ref. ^34^. The particle dynamics occur in a 3D Cartesian coordinate system. In order to update the light intensity distribution using the discretized, axisymmetric paraxial Helmholtz equation (Eq. 1), the particle density component is calculated by mapping the particle positions to a cylindrical coordinate system. It then follows that the optical forces on the particles are mapped from a cylindrical to Cartesian coordinate system for the particle velocity and position update.

The light is initialized as a diverging, i.e., convex wavefront, Gaussian beam with a waist of 1 m. The particle beam, composed of lithium atoms, is also initialized with a radius of 1 m at the injection plane, i.e., $z=0$ m. These particles are injected with a drift velocity of 0.1c and temperature of 0.1 K. For these conditions, a laser detuning frequency of $-\,19$ THz is used to maximize the effective trapping force. 100 computational macroparticles are injected every time step, and the macroparticle weight is defined by the desired injection density. The total number of macroparticles is 90,000–180,000, taking approximately 3 h on a single core CPU to reach steady state, which is monitored by the changes of the number of macroparticles in the calculation domain. The computational domain spans 5 m radially and approximately $10z_r$ axially, with 2000 cells in both directions. Results are obtained by averaging the light intensity and particle density distributions over 1000 iterations, once the simulation reaches steady state.

### Optimal configuration

From a given beam waist, particle temperature, and light detuning frequency, the mutual propagation of the coupled particle-light beam is studied varying the laser power, $P_0$, and particle density at injection, $N_{\mathrm{inj}}$. After conducting parameter sweeps on the light power and injected particle density in the next two sections, the optimal conditions are found to be 2.2 TW light power and $5.0\times 10^{13}$ m$^{-3}$ particle density, which is close to the critical density for a single mode waveguide, $N_0$.

**Figure 5:**
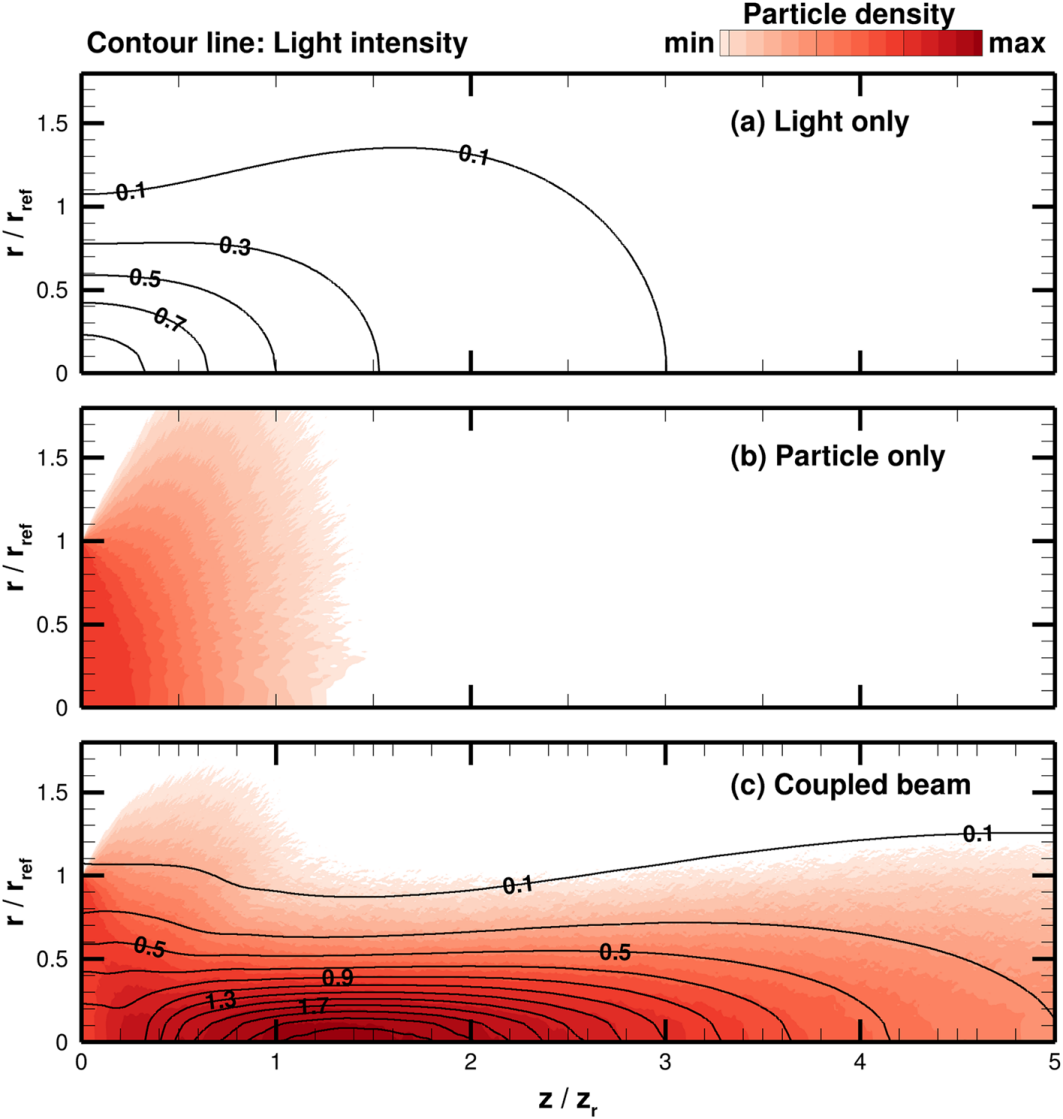
Comparison of optimized, coupled beam results with vacuum cases: (**a**) light intensity for a light beam propagating in vacuum, (**b**) particle denisty for a particle beam propagating in vacuum, and (**c**) light intensity and particle density for a coupled light-particle beam. The light intensity and particle density are normalized to their corresponding center line values at injection, i.e., $z = 0$. Min and max values of the color contour correspond to 0.1 and 3, respectively.

Figure 5 shows the results of the optimized coupled beam compared with the light and particle beam propagating independently in vacuum, i.e., diffracting light beam and diffusing particle beam. The light intensity and particle density distributions are normalized to the initial centerline intensity and injection beam density at $r=0$ and $z=0$ for the decoupled beam cases, respectively. The results clearly show how effective particle trapping and waveguiding maintain a collimated coupled beam for several Rayleigh ranges further than the decoupled beams. The coupled beam also shows regions of intensity and density that are larger than their injection values, illustrating that the particle-light beam is trapped and focused. These concentrated areas could provide additional thrust to the space probe if used for space propulsion applications. It is important to note that these simulations assume an idealized model of the light beam in which the chosen frequency and amplitude remains constant, while in realistic systems there can be uncertainties and noise. For instance, shifts in the laser frequency may impact the coupled beam results. The focus of this paper is to demonstrate the coupling between idealized light and particle beams. Investigation of the effects of realistic uncertainties and noise on the beam propagation is reserved for future work.

### Effects of the particle density

One key parameter for the coupled system is the injected particle beam density. From optical waveguide theory, a relation was determined for a critical density that would maximize light waveguiding for single mode propagation, as discussed in Eq. (11). In order to confirm the implications of this relation and analyze the effect of multi-mode light on propagation distance (assuming axisymmetric beams), the injected particle density is varied in this section. The light power is fixed to 2.5 TW and the other parameters are the same as the previous subsection.

**Figure 6:**
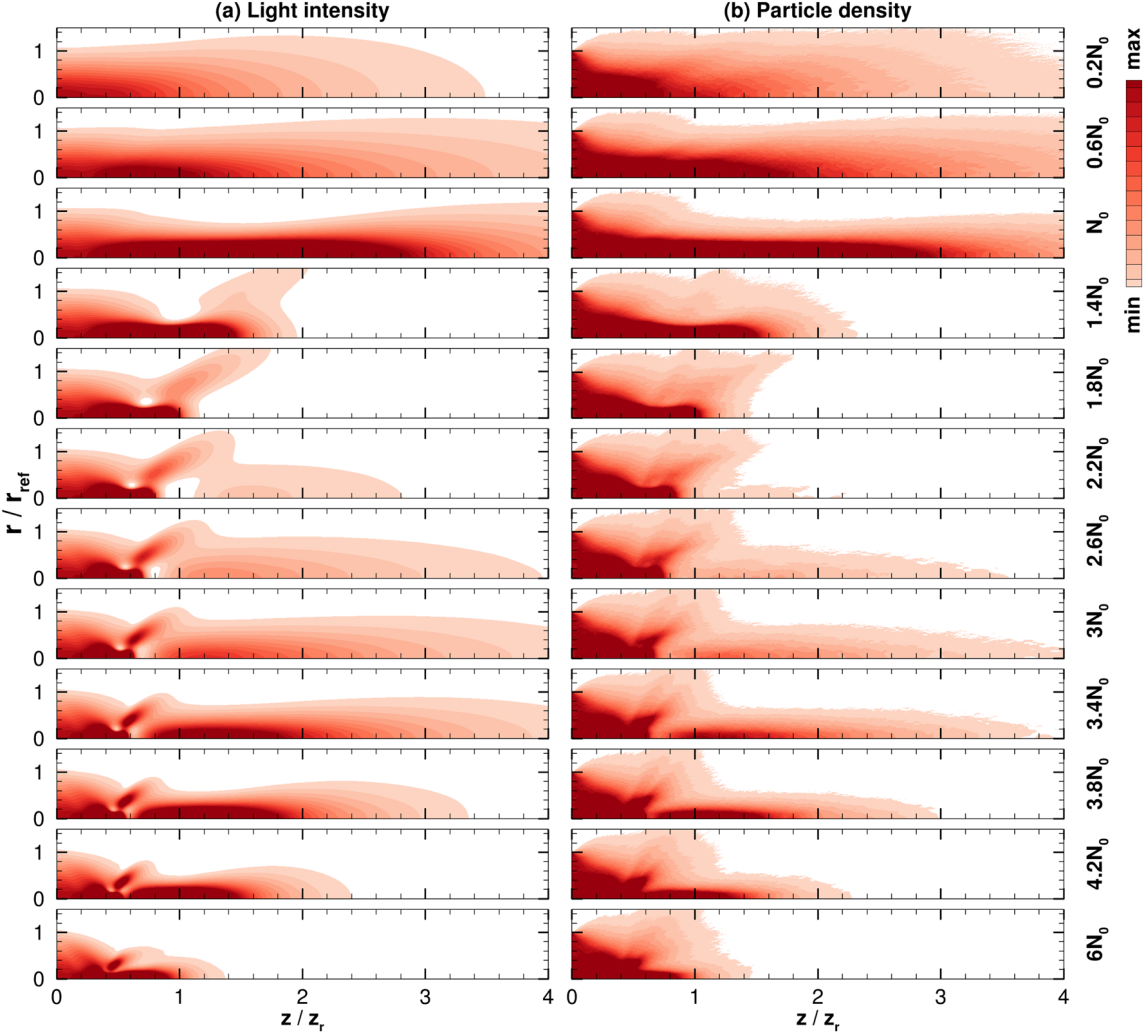
Normalized (**a**) light intensity and (**b**) particle density over a range of injection particle densities relative to $N_0 = 5.0\times 10^{13}$ m$^{-3}$, which is the critical density that results in a single mode waveguide, i.e., $V=2.405$. The min and max values are 0.1 and 1, respectively.

Figure 6 shows the normalized laser intensity and particle density for the coupled system from simulations over a range of injection particle beam density that covers the single mode and the first two higher order mode cutoffs (V = 2.405, 3.832, and 5.136), which corresponds to the particle density of $N_0, 2.5N_0,$ and $4.5N_0$, respectively. Note $N_0 = 5.0\times 10^{13}$ m$^{-3}$. When $N_{\mathrm{inj}}<N_0$ the beam shapes are similar to vacuum propagation which shows some noticeable trapping and guiding as $N_{\mathrm{inj}}$ approaches $N_0$. The $N_0$ case, which is approximately the density for the single mode shows the most collimated beam for both particle and light. This clearly shows the ideal parameter choice for particle density and confirms the relations from waveguide theory. However, additional testing revealed that the optimal density was actually slightly lower than the critical density from waveguide theory. The case that prolonged the beam propagation distance has a V-parameter of 2.3 instead of $V=2.405$ that the theory of single-step fibers predicts. This discrepancy can be attributed to the fact that the particle beam is changing and therefore does not match the influence of a fixed waveguide. The higher densities show a shorter propagation distance. This is most likely due to the larger index of refraction gradient resulting in larger refraction angles for the light. The light beam overfocuses and influences the particles, leading to a pinched beam effect that will be discussed in a later subsection. Around the second mode, $N_{\mathrm{inj}} = 2.6N_0$, the beams begin to show signs of improved trapping and guiding after $z=z_r$, after the beams go through overfocusing. This can be attributed to the fact that the particle density in this secondary region is now similar to the density for the single mode waveguiding of the reduced beam. It is interesting to note that this secondary region of guiding and trapping shows another co-propagation of light and particle beams around $N_{\mathrm{inj}} = 3.4N_0$ before decaying at larger injection densities.

Overall, Fig. 6 illustrates how the beam shape appears optimal around the single mode, and the formation of radial intensity profiles that match the higher order modes are mitigated by the dynamic nature of the system. In other words, since the waveguide theory assumes that the waveguide is a fixed medium of constant refractive index, the expected results for higher modes become less likely as the particle beam deviates from the fixed waveguide it is meant to represent. However, at certain axial locations in the higher density cases, the intensity radial profiles appear to show higher mode shapes. The numerical results suggest that the multimode structure can be an onset of axial dynamics that produces a secondary particle-light self-guided beam propagation.

### Effects of the laser power

**Figure 7:**
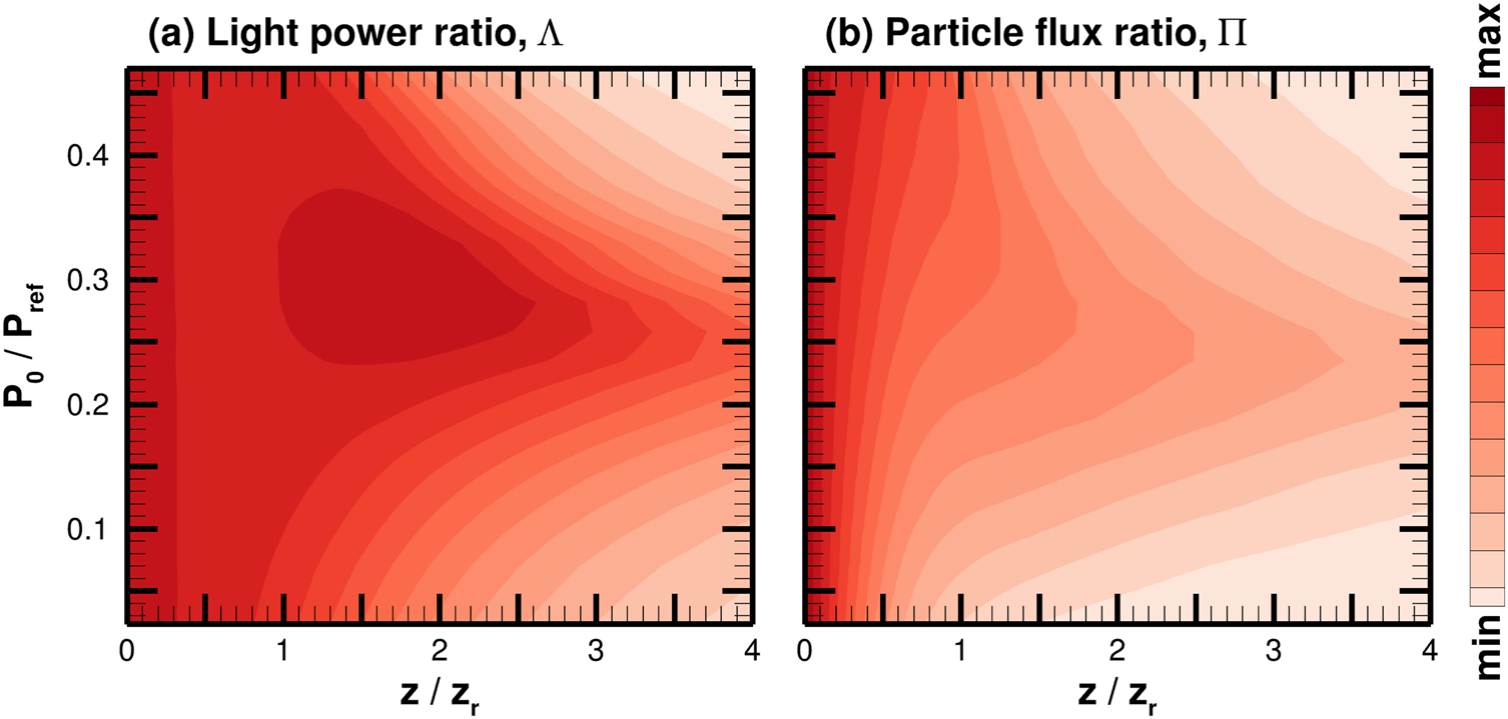
(**a**) Light power ratio and (**b**) particle flux ratio for a range of injected laser power. Min and max values correspond to 0.1 and 1.0, respectively. The injected power is normalized by $P_{\mathrm{ref}}=8.52$ TW.

Parametric studies of the mutually guided particle-light beam propagation varying the light power is performed with all other parameters fixed. In order to quantitatively assess the optimized beam propagation distance, light power ratio is defined as $\Lambda (z)=\int ^{w_0}_0 2\pi rI(r,z)\mathrm{d}r/P_0$, which essentially describes how much light power resides within the initial beam waist area at a specified axial distance relative to the light power at injection. Figure 7a illustrates how the light power ratio varies for different cases of injected light power along the axis of propagation. Note the injection laser power is normalized by $P_{\mathrm{ref}}=\pi r_{\mathrm{ref}}^2I_{\mathrm{ref}}/2=8.52$ TW. The result in the limit of $P_0/P_{\mathrm{ref}} \rightarrow 0$ corresponds to the solution of a natural diffraction as the effective trapping force approaches zero (see Eq. 7), i.e., no particle trapping. On the other hand, the results at high laser intensities (e.g., $P_0/P_{\mathrm{ref}} \ge 0.5$), the solution also shows limited beam propagation due to strengthened scattering forces and an over-focusing effect. In order to investigate the relative impact of these loss mechanisms, the over-focusing effect is discussed in the next subsection. Due to the balance between the effective trapping and the loss mechanisms, for an intermediate range of injected laser power, the light intensity is maintained for a large distance, where the particle and light beams self-guide each other. The maximum propagation is observed around $P_0=2.2$ TW ($P_0/P_{\mathrm{ref}}=0.26$). This observation is in agreement with the expression, $P_{opt}=2\pi w_0^2c\varepsilon _0k_BT_\perp /\alpha '$, which is the same formulation as $P_{\mathrm{ref}}$ except it uses the simulation rather than reference values. The light power ratio is defined as the power within the initial beam waist, $w_0$. Thus, the decrease in $\Lambda$ around $0.5z_r - z_r$ for $P_0/P_{\mathrm{ref}} = 0.23 - 0.35$ is due to the light initially diffracting. The $\Lambda$ exceeding its injection value at $z_r - 2.5 z_r$ indicates that the outward radiating light beam is entrained into the center of the beam due to waveguiding effects.

Similarly for the particle beam, particle flux ratio is defined as $\Pi (z)=\int ^{w_0}_0\Gamma (z)rdr/\int ^{w_0}_0\Gamma (z=0)rdr$, which describes the particle flux within the initial beam cross-sectional area relative to the injected particle flux. Since the axial forces on the particle have minimal impact on its injected drift velocity, this flux ratio could be considered the particle beam power ratio, where the particle flux is replaced with the particle kinetic energy flux. The results for the particle flux ratio are exhibited in Fig. 7b. As some fraction of the particles are lost initially due to the radial temperature being finite, the integrated particle flux is observed to be always smaller than the injected particle flux, hence $\Pi <1$. However, similar to the light power ratio $\Lambda$, the integrated particle flux shows a smaller loss around $P_0/P_{\mathrm{ref}}= 0.2 - 0.3$. The maximum distance for particle beam propagation is found at a slightly lower power case than for the light power ratio, as shown in Fig. 7. This is likely due to the definition of $\Lambda$ and $\Pi$, integrating the light intensity and particle flux density within the initial beam waist, $w_0$. The particles are lost in the radial direction due to their thermal motion more than the photons within the first Rayleigh range.

### Particle loss due to over-focusing effects

In the previous subsections, mutually-guided propagation of the particle and light beam is observed. The particle-light coupled simulation shows that the self-guided propagation is maximized when the single mode waveguiding is achieved. However, the self-guiding performance drops at larger particle density and larger light intensity. It can be considered that such optimality results from the balance between the trapping (due to dipole force and waveguiding) and the loss (due to scattering force).

In this section, the simulations are performed without the scattering force exerted on the particles to investigate whether detrapping due to the scattering force is the only loss mechanism for the self-guided beam. In the absence of scattering force, the effective trapping force is always positive (as discussed in Fig. 3), which could result in an infinitely long propagation of the light and particle beams. In addition, the mutual trapping would be more effective as a larger light intensity is considered as $U_{max} \gg k_B T_\perp$. The following simulation considers a hypothetical situation where the dipole force is only considered in addition to the waveguiding of light beam due to the change in refractive index, by removing particle-photon scattering and setting the injected particle temperature to zero. Thus, the particle beam is initialized as an ideal, collimated beam that, in the absence of other forces, would propagate infinitely. The particle beam density at injection is assumed to be $N_0=5.0\times 10^{13}$ m$^{-3}$.

**Figure 8:**
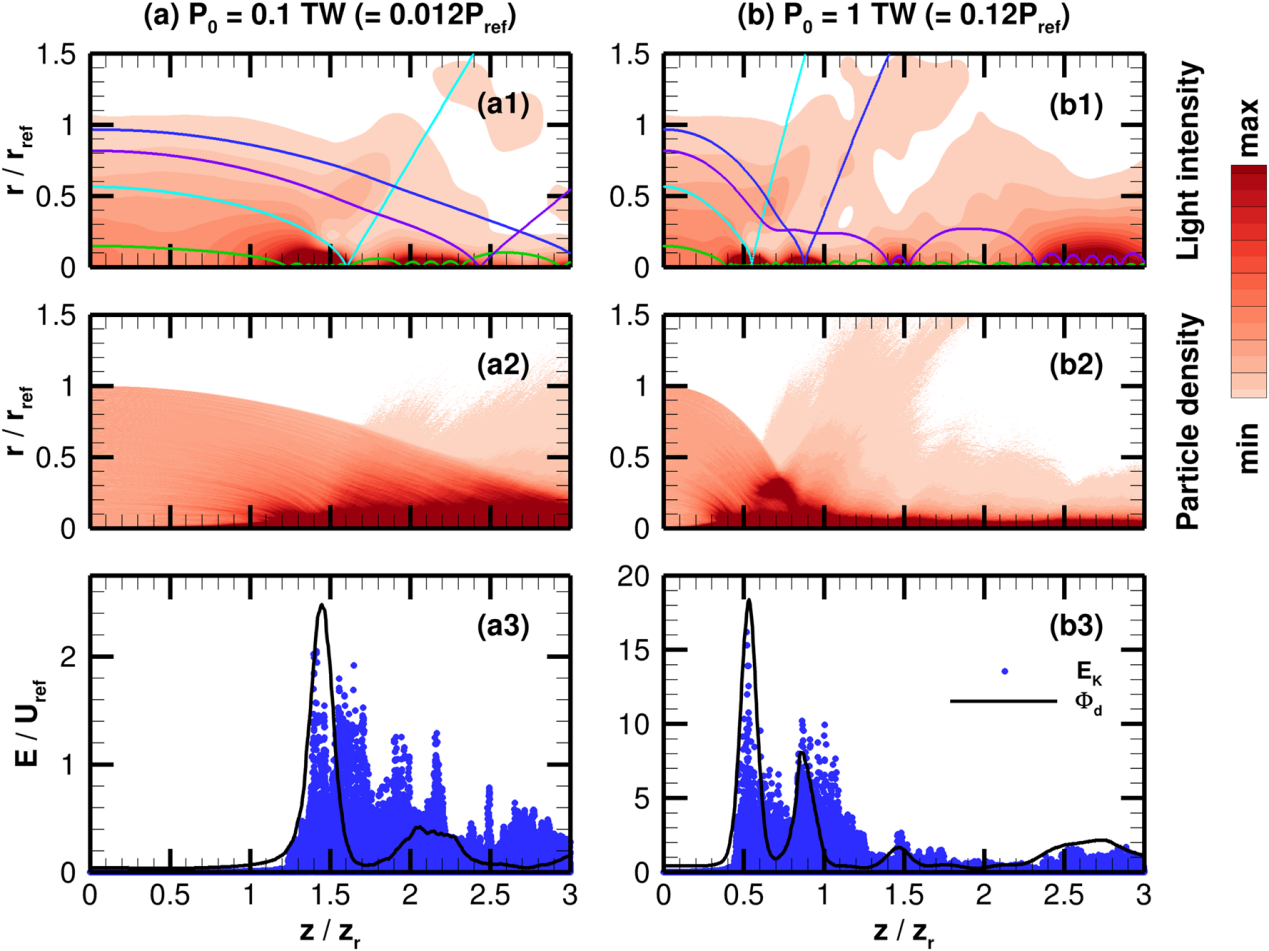
Beam propagation in the absence of the scattering force, i.e., only dipole force is considered, and zero particle temperature for a light beam power of (**a**) 0.1 TW and (**b**) 1 TW. The over-focusing effect is demonstrated through (a1, b1) the normalized light intensity with four representative particle trajectories and min and max values corresponding to 0.1 and 2.5, (a2, b2) the normalized particle density with min and max values corresponding to 0.1 and 4, and (a3, b3) the radial kinetic energies, $E_K$, of the particles compared with the depth of the light potential well, $\Phi _d$, at each axial location. The energies, *E*, are normalized by $U_{\mathrm{ref}}=2.76\times 10^{-24}$ J.

Figure 8 shows the results of a coupled beam assuming two laser powers at 0.1 TW and 1 TW ($P_0/P_{\mathrm{ref}} = 0.012$ and 0.12, respectively). Despite only having the dipole force on the particles, i.e., the scattering force is not accounted for, the light intensity and particle density distributions from Fig. 8a1,a2, respectively, show considerable losses. By analyzing these results, the cause of these losses can be described through a connected sequence of events due to overfocusing. First, the strong dipole force causes the particles to be accelerated towards the centerline of the beam, which for an axially constant dipole potential structure (such as the one visualized in Fig. 1) would not present a problem. However, since this is a fully coupled system, amplification of the particle density guides the light beam to further converge and cause a concentrated region of high light intensity. This region of high light intensification creates a much stronger dipole force, i.e., a pinching effect, that increases the particle speed in the radial direction. This can be seen in Fig. 8a3, which shows the phase plot of radial (transverse) kinetic energies of the particles along the axis of propagation. The increase in the optical dipole potential that leads to particle trapping is demonstrated by tracking the depth of the dipole potential structure. This depth is defined as

12 $$\begin{aligned} \Phi _d(z)=\frac{\alpha 'I_{max}(z)}{2c\varepsilon _0}, \end{aligned}$$

where $I_{max}(z)$ is the maximum intensity value at the given axial location. Here, the particle kinetic energy and potential depth are normalized by $U_{\mathrm{ref}}=2k_BT_{\mathrm{ref}}=2.76\times 10^{-24}$ J. Initially the dipole potential increases followed by the increase in the particle kinetic energy. Following this intensified region, the highly accelerated particles cross through the centerline region of the beam, i.e., $r=0$, as can be seen from the particle trajectories in Fig. 8a1 and travel into an axial location in which the light beam is not deeply intensified due to the multidimensional propagation of the beam. The drop in potential depth of the light beam after $1.5z_r$ creates a disparity that allows for substantial, high-energy particles to escape the dipole potential. The particles lost in the radial direction make the particle beam density lower, which consequently reduces the amount of light being guided.

The example particle trajectories in Fig. 8a1 further exemplify the over-focusing effect. A particle injected near the edge of the beam (represented by the dark blue line) is accelerated towards the centerline of the beam. As illustrated by the purple and light blue lines, the next two trajectories of particles closer to the center of the beam are drawn towards the centerline earlier in the domain. This is expected since these particles are initialized closer to the centerline. After a particle crosses through the center of the light beam, it appears to ballistically propagate out of the beam. Lastly, the green line trajectory shows that a particle injected close to the center of the beam is deeply trapped within the potential well, resulting in a regular bouncing motion for the particle path.

Figure 8b shows the results when increasing the laser power to 1 TW, which illustrates that the pinching effect scales with the strength of the dipole force. The convergence of the particle beam occurs earlier in the domain, indicating that the pinching effect is much stronger. It can be seen in Fig. 8b1 how the particle trajectories change for the higher light intensity. The blue line marks how the location at which the beam is pinched occurs at a shorter axial distance. The light blue and green trajectories also illustrate how the path is simply modified to reflect a stronger dipole force. The path of the particle from the purple line, however, is significantly altered by the irregularity of the potential structure. Throughout its journey, the particle is accelerated and decelerated in a manner that results in a lower velocity around the centerline, which causes the particle to become quasi-trapped. Therefore, the injected particles travel in the domain depending on the path they take through the multidimensional structure of the light intensity. Light intensity and particle beam density, as shown in Fig. 8b1,b2, respectively, show the complex nature of the particle-light coupling. The particles are deeply trapped around the centerline. However, the mechanism at which the particles detrap from the light beam is clearly shown in Fig. 8b3, similar to Fig. 8a3.

While it was initially hypothesized that the lack of particle-photon scattering can be beneficial to the particle-light self-guided propagation, the simulation results in this subsection show that the pinching effect (due to the multidimensional nature) without any scattering mechanism results in a significant loss mechanism. This effect is especially influential considering that the particle beam is initialized with an infinite characteristic length, $z_p$. Hence, with the demonstration of the over-focusing effects in this section, it can be concluded that the optimal mutual guiding between particle and light beams is achieved by not only minimizing the scattering force, but also is related to the multidimensional nature of the particle-light coupled beam propagation. The optimal mutual guiding occurs when the particle trapping is strong enough to overcome particle-photon scattering and thermal diffusion without being so strong that it leads to the over-focusing effect.

## Conclusion

A fully verified and validated code is used to explore the feasibility of the mutual guiding of an initially expanding particle beam and an initially diffracting light beam. The simulation conditions chosen are selected based on the application for the deep space propulsion, but the nonlinear physics shall be scalable for different applications. The relevant parameters that dictate the propagation distance of the coupled beam are identified and optimized theoretically and computationally.

Results indicate that the light detuning that maximizes the dipole force with respect to the scattering force can be determined once the beam waist is chosen. The simulation results show that propagation distance is maximized by using a particle density approximately equal to the critical density for a single mode waveguide. It was observed that an optimal light power is needed to maximize the nonlinear coupling, overcoming scattering and particle loss due to its thermal motion. At higher light power, the large scattering force and the pinching effect due to the large dipole force can lead to reducing the self-guiding effects of the particle and light beams. Paths to further optimize the beam will be to focus on the choice of beam waist and beam shape, which affects the strength of the dipole force, as well as the characteristic lengths of the beam. Nevertheless, the simulation result of the optimized, coupled beam clearly shows that the propagation distance can be made larger than the Rayleigh range, shedding light into manipulation of light and particle beams.
